# The Effect of Adding Different Levels of Curcumin and Its Nanoparticles to Extender on Post-Thaw Quality of Cryopreserved Rabbit Sperm

**DOI:** 10.3390/ani10091508

**Published:** 2020-08-26

**Authors:** Sameh A. Abdelnour, Mahmoud A. E. Hassan, Amer K. Mohammed, Ahmad R. Alhimaidi, Naif Al-Gabri, Khalid O. Al-Khaldi, Ayman A. Swelum

**Affiliations:** 1 Department of Animal Production, Faculty of Agriculture, Zagazig University, Zagazig 44511, Egypt; 2 Animal Production Research Institute, Dokki, Giza 12619, Egypt; m.hassan55213@gmail.com; 3 Department of Animal Production, Faculty of Agriculture, Mansoura University, Mansoura 35516, Egypt; amer.kamal@gmail.com; 4 Department of Zoology, College of Science, King Saud University, Riyadh 11451, Saudi Arabia; AhmedAl-Himadi@ksu.edu.sa; 5 Department of Pathology, Faculty of Veterinary Medicine, Thamar University, Dhamar 2153, Yemen; naifaljabry@yahoo.com; 6 Laboratory of Regional Djibouti Livestock Quarantine, Abu Yasar international Est. 1999, Djibouti; 7 Royal Oman Police, Mounted Division, Veterinary Section, Muscat 113, Oman; khalidkhalid@gmail.com; 8 Department of Animal production, College of Food and Agriculture Sciences, King Saud University, P. O. Box 2460, Riyadh 11451, Saudi Arabia; 9 Department of Theriogenology, Faculty of Veterinary Medicine, Zagazig University, Sharkia 44519, Egypt

**Keywords:** rabbit, cryopreservation, semen, redox status, apoptosis, nano-curcumin

## Abstract

**Simple Summary:**

In rabbit farms, artificial insemination is usually accepted using semen preserved around 18 °C. However, the use of cryopreserved rabbit semen is limited, due to excess oxidative stress and produce sperm dysfunction. The advancements in nanotechnology tools have allowed molecular-based targeting of cells through effective, safe, and biocompatible magnetic nanoparticles with promising potentials in reproductive sciences. In these regards, the current work aimed to explore the potential role if the effect of curcumin nanoparticles supplementation in semen extender on post/thawed rabbit sperm quality. Results revealed that the CUNPs (1.5 µg/mL) showed superior enhancements impacts for the post-thawing sperm motion and redox status, as well as a significant reduction in apoptotic and necrotic sperm cells. This confirmed the constructive application of nanoparticle to enhance the cryopreserved rabbit’s sperm function.

**Abstract:**

The cryopreservation process adversely affects sperm function and quality traits, causing some changes at biochemical and structural levels, due to mechanical, thermal, osmotic, and oxidative damage. Supplementation with curcumin nanoparticles could prevent and even revert this effect and could enhance the post/thawed sperm quality in the rabbit. The study amid to explore the effect of curcumin (CU) and curcumin nanoparticles (CUNPs) supplementation in semen extender on post/thawed rabbit sperm quality. Twelve fertile, healthy rabbit bucks were included, and the ejaculates were collected using artificial vaginas. Rabbit pooled semen was cryopreserved in tris-yolk fructose (TYF) extender without any supplement (control group) or extender supplemented with CU at levels of 0.5, 1 or 1.5 µg/mL (CU0.5, CU1.0, and CU1.5, respectively) or CUNPs at levels of 0.5, 1, 1.5 (CUNPs0.5, CUNPs1.0, and CUNPs1.5, respectively) and was packed in straws (0.25 mL) and stored in liquid nitrogen (−196 °C). Results revealed that CUNPs1.5 had a positive influence (*p* < 0.05) on post-thawing sperm progressive motility, viability, and membrane integrity as compared with the other groups. Percentages of dead sperm, abnormalities, early apoptotic, apoptotic, and necrotic sperm cells reduced (*p* < 0.05) in CUNPs1.5 as compared to other treatments. Using 1.5 µg/mL of CUNPs significantly improved total antioxidant capacity (TAC), GPx, while MDA and POC reduced (*p* < 0.05) in CU1.5 in comparison with other groups. SOD values were enhanced (*p* < 0.05) in CUNPs1.0 and CUNPs1.5 in relation with other treatments. Conclusively, the addition of curcumin and its nanoparticles to the extender can improve the post-thawed quality of rabbit sperm via redox signaling and reduce the apoptosis process.

## 1. Introduction

Gamete cryopreservation is a critical system for artificial insemination (AI) that permits cheap, the global distribution of gametes with superior genetics. In the last decades, the application of artificial insemination in the rabbit industry has been received global attention in countries where an intensive system for rabbit breeding is practiced, particularly in Europe [1]. Studies have indicated higher fertility rates and prolificacy were employed with cooled semen for a short time (36 h) [2]. In the other side, AI with cryopreserved rabbit sperm had inferior prolificacy or fertility than fresh or cooled semen [1]. Cryopreservation technique is recognized to impair sperm in a multiplicity of means resulting in sub-lethal impairment and alterations responsible for a shortened lifespan of sperm cells [3].

Several stresses factors affecting sperm function during the process of cryopreservation, such as decline in temperature, elevated osmolality, the addition of cryoprotectants, ice formation, and generation of oxidative stress (OS) [4]. The excess of OS production throughout the freezing/thawing process modifies protein and lipid structure, reduction viability, and motility; cause loss to mitochondria, acrosomes, tails, and enhance DNA fragmentation in sperm [1,5]. Besides, the freezing and thawing procedures prompt rearrangement of lipid membranes, resulting in increased intracellular calcium and fluidity, which ultimately initiates precipitation, triggering several instabilities of cell physiology [5,6,7]. It is accepted that sperms are mainly vulnerable to damage caused by OS, because of its rich in polyunsaturated fatty acids that readily undergo peroxidation, alter redox signaling, making secondary products that can oxidize sperm lipids and proteins [3,8]. Antioxidant supplementation to the freezing medium, for counteracting OS and sustainability of membrane integrity during semen freezing, has earlier been termed in several animal species [7,9,10,11].

Curcumin is an important natural polyphenolic compound, which has been broadly applied for multimodal uses, such as cosmetic agents, food processing, and in some medical preparations. Curcumin has been reported to be effective in improving sperm mobility in asthenozoospermia patients [12], owing to its antioxidant capacity, and free radical scavenging [12,13]. Due to its safety, low toxicity and efficacy use in human and animals’ experiments, the US Food and Drug Administration has recommended curcumin as “generally regarded as safe” [14]. However, curcumin was limited in medical applications, due to its low bioavailability, and solubility, which might be caused by poor absorption and rapid metabolism [15]. To overcome this complication, on the one hand, different formulations of developed curcumin nanoparticles have been explored. 

The nanotechnology sector has progressed and expanded vastly in the past years, with different potential applications. Nanoparticles are efficient approaches interacted with biological systems because of their nanoscale and large surface area. It was confirmed that the bioavailability and absorption of some drugs were considerably improved when managed by nanoparticles [11]. Reports exhibited that the use of nanoparticles can enhance semen quality and freezability by decreasing the OS and protection the spermatozoa from cryo-damage [7,11,16,17,18]. Although, several previous studies have demonstrated that curcumin nanoparticles (CUNPs) could improve sperm kinetics, motion characteristics and testis function subjected to the induced OS in cryopreserved spermatozoa of different mammalian species [12,15,18,19]; however, no previous report explore the protective role of CUNPs in cryopreserved rabbit semen. Moreover, studies of cryogenic injuries and ultrastructure of spermatozoa are important for understanding the machinery effects on sperm cryogenic, and thus, enhancing cryopreservation procedures. 

Due to highly bioavailability, more stable and small scale of CUNPs, we hypothesized that the use of nanoparticles of curcumin could help to protect cryopreserved sperm from negative impacts of OS produced during cryopreservation process in a rabbit model. Therefore, the current study is conducted to assess the effects of curcumin nanoparticles (CUNPs) added to the freezing medium of rabbit semen on post-thawing sperm features, chromatin integrity, ultrastructure alterations and apoptosis of spermatozoa and redox status in seminal plasma.

## 2. Materials and Methods

This research was implemented at the Rabbit Farm of Animal Production Department, Faculty of Agriculture, Mansoura University, Egypt, in cooperation with electron microscope unit (EM-Unite Mansoura University). All experimental protocols were consistent with the European guidelines with respect to the care and use of experimental animals, and the experimental protocol was performed following approval by the Animal Care Committee and Animal Research Ethics Mansoura University, Egypt.

### 2.1. Characteristics of Curcumin Nanoparticles

Curcumin nanoparticle (CUNPs) and Curcumin (CU) were purchased from Nano Gate Company (Nasr City, 11765, Cairo, Egypt). Curcumin nanoparticle (CUNPs) mean size, polydispersity index (PDI) and Zeta potential were assessed using a Zetasizer Nano series (Nano ZS90 Malvern Instruments Ltd., Worcestershire, UK) according to photon correlation spectroscopy method. The rates of PDI are ranged (0–1); an upper rate showed a fewer homogeneous nanoparticles size distribution. As the magnitude of the zeta potential provided a reference of the possible constancy of the colloidal structure. Then, the mean particle size, PDI and zeta potential were ready to evaluate in the different levels of CUNPs [20]. Moreover, the morphology of CUNPs was considered by transmission electron microscopy (TEM) on an electron microscope (at 200 kV; JEOL-JEM-2100) according to the method of Chen et al. [21].

### 2.2. Animals and Semen Collection

In the present study, twelve healthy bucks’ rabbits (10–12 months and 3.6 ± 0.2 kg of body weight) were selected. Bucks were reared under similar management circumstances and environmental situations. Animals were independently kept in slandered cages (60 × 40 × 35 cm) and inside an open-system type farm (uncontrolled system). The bucks received a basal diet formulated to cover the nutrient requirements [22]. The animals were trained for the semen collection two weeks before the beginning of the trial using an artificial vagina. A mature female was used as a mount and introduced into the buck for semen collection. Ten ejaculates were collected from each buck in a rate of one ejaculate/week for 10 successive weeks. The collected semen samples were assessed microscopically after removing gel clot, and only good ejaculates that showed anterior forward progressive motility more than 70% were pooled to eliminate the individual’s variations and included in the current study. 

### 2.3. Semen Processing and Experimental Design

The tris yolk fructose (TYF) diluent was prepared according to the methods of Di Iorio et al. [23]. Concisely, 1.675 mg/dL Citric acid anhydrous, 3.028 mg/dL Tris and 1.25 mg/dL Fructose were dissolved in 100 mL distillated water. Then, 7% (*v/v*) glycerol and 20% (*v/v*) egg yolk were added. Finally, 100 µg/mL streptomycin and 100 IU/mL penicillin were added. The following supplements were added to the extender to explore their impacts on sperm cryopreservation. In the control group, the extender was not supplemented by any additive. In the CU0.5, CU1.0 and CU1.5 groups, the extenders were supplemented with 0.5, 1 or 1.5 µg/mL curcumin, respectively. In the CUNPs0.5, CUNPs1.0 and CUNPs1.5, the extenders were supplemented with 0.5, 1 and 1.5 µg/mL nanoparticles curcumin (CUNPs), respectively. 

The pooled semen sample was divided equally into seven parts. Each part was gradually diluted 1:4 with one of the previously prepared extenders at 37 °C. Treated diluted semen was gradually cooled and retained at 4 °C for 4 h as an equilibration period, then packed in French straws (0.25 mL, IVM technologies, L’ Aigle, France), located 4 cm overhead liquid nitrogen for 10 min then kept in liquid nitrogen (−196 °C) for one month conferring to the protocol of Salisbury et al. [24]. 

### 2.4. Assessment of Post-Thawed Sperm Quality

#### 2.4.1. Sperm Progressive Motility and Plasma Membrane Integrity 

The frozen straws were thawed in a water bath at 37 °C for 30 s. Sperm progressive motility (%) was examined by phase-contrast microscope (Leica DM 500) supported with a hot plat adjusted to 37 °C [24]. In order to explore efficient plasma membrane of spermatozoa, hypo-osmotic swelling (HOST) assessment was performed as termed by Caycho et al. [25]. In brief, a 10 µL of semen was incubated with 100 µL hypo-osmotic solution (3.67 g/L sodium citrate and 6.75 g/L fructose, to offer osmolality level of 75 mOsmol/L) at 37 °C for 30 min. Then, a 10 µL of the mixture was employed on a microscope slide and fixed with a cover slip. Three hundred sperms were evaluated under phase-contrast microscopy (Leica DM 500) at 400×. Sperms with swollen and coiled tails were considered to have intact plasma membrane.

#### 2.4.2. Sperm Viability and Abnormalities

The sperm viability and abnormalities were evaluated using the eosin (1.67%) and nigrosin (10%) stain technique. Briefly, a 10 µL of sperm sample and 20 µL of stain were mixed well on a prewarmed slide and directly expanded with another slide. Under a light microscope (Leica DM 500), three hundred sperms were examined in each sample (400×). The number of dead sperms (red stained) and the number of sperms bearing morphological abnormalities were recorded [26].

#### 2.4.3. Sperm Apoptosis

Samples of spermatozoa were prepared for Annexin V staining according to the method of Chaveiro et al. [27], with certain adjustments. Concisely, one mL of sperm suspensions was supplemented to 2 mL binding in 5 mL tube. Next, 100 µL of sperm suspensions were transferred in another 5 mL test tube included with 5 µL of annexin V (A) and directly 5 µL propidium iodide (PI) staining, and then samples were incubated at room temperature for 15 min in dark conditions. After that, sperms were suspended in 200 µL binding buffer. The sperm samples are ready for flowcytometric assessment. 

Flowcytometric examination was implemented on Accuri C6 (BD Biosciences, San Jose, CA, USA) Cytometer provided with Accuri C6 software (Becton Dickinson) for analysis and acquisition [28]. The platelet calculating was prepared by the BD Accuri TM C6 flow cytometer. Different classes of spermatozoa were defined according to [29]. The A/PI system shown four sperm subpopulations, two PI negative (either A− (alive) or A+ (apoptotic); and two PI positive (dead cells), either A+ (dead, late apoptotic or early necrotic cells) or A− (dead, late necrotic cells). 

#### 2.4.4. Ultrastructure Changes of Spermatozoa

Semen samples were managed for transmission electron microscopy (TEM) assessment based on the method of Oliveira et al. [30], with minor changes. In brief, samples (5 straw) were exposed to centrifugation and suspended in a fixative solution consisted of 2.5% glutaraldehyde in phosphate-buffered saline for 2 h at 4 °C, and then kept in osmium tetroxide (1%) at room temperature for one hour. The samples were dehydrated in an ethanol gradient, treated with acetone and embedded in ultrathin-sectioned (60–70 nm) and Epon resin (Epon 812; Fluka Chemie, Switzerland). Ultrathin sections in the cells were identified by TEM (JEOL-JEM 2100 at 80 KV). The ultrastructure of spermatozoa was surveyed in 100 spermatozoa each group.

#### 2.4.5. Biochemical Evaluation of Antioxidants Indices

Post-thawed semen samples were centrifuged for 15 min at 1500 rpm at 4 °C (Sigma 2–16 KL), then the supernatant extender was separated and stored at −20 °C pending analysis. By thiobarbituric acid reaction technique, the malondialdehyde (MDA) level in the extender was measured [31]. The activities of glutathione peroxidase (GPx) and superoxide dismutase (SOD) were detected by commercial enzyme detection kit (MyBioSource, Giza, Egypt) according to the manufacturer’s protocol. The total antioxidant capacity (TAC) was assessed by checking hydrogen peroxide decomposition using OxiSelect commercial kits (Cell Biolabs, USA; [11]. Protein carbonyls were used for protein oxidation in the extender, detection of total protein carbonyls was performed using a commercial protein carbonyl ELISA kit (MBS2600784; MyBioSource, Giza, Egypt) based on the manufacturer’s procedure [3].

### 2.5. Statistical Analysis

The normality of data distribution was evaluated in the Shapiro–Wilk W test. Data were analyzed using analysis of variance (ANOVA) by one-way design, using General Liner Model (GLM) procedures of SAS [32]. The significant differences among means at probability ≤0.05 were examined by Duncan’s Multiple Range Test [33]. The data were presented as mean ± standard error means (SEM).

## 3. Results

### 3.1. Characteristics of Curcumin Nanoparticles (CUNPs)

**Figure 1:**
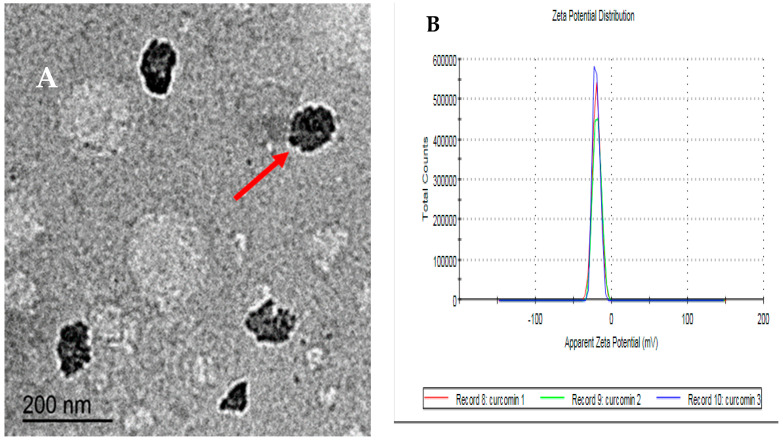
A transmission electron microscopic (TEM) image showing a nearly spherical shape of curcumin nanoparticles (CUNPs) with a smooth surface and uniform size distribution (**A**) and histogram showing the apparent zeta potential (mV) of CUNPs (**B**).

In order to confirm the synthesis of curcumin nanoparticles (CUNPs), the efficiency testing of CUNPs using Zeta potential was performed (Figure 1). Further depiction of CUNPs was performed by TEM to determine the average size. From the TEM analysis, the mode value was found with the average size of 100 nm (Figure 1A). The zeta potential of CUNPs was found to be −25 mV which presented strong stability of CUNPs (Figure 1B).

### 3.2. Effect of CU and CUNPs on Sperm Characteristics in Post-Thawed Rabbit Semen

The influence of CU and CUNPs on the percentages of progressive motility, membrane integrity, dead sperm (%) and abnormality are presented in Table 1. The progressive motility and membrane integrity percentages were significantly (*p* < 0.05) enhanced in post-thawed rabbit semen diluted with extender incorporated with CUNPs at a dose of 1 and 1.5 µg/mL when compared with the other treated groups and the control, is the best with 1.5 µg/mL. Following by CUNPs1.5, a higher number of cell sperms with integrity cell membrane was detected in the CUNPs1.0 and CU1.5. The greatest (*p* < 0.01) percentage of abnormal forms and dead sperm were observed in the CUNPs1.5 group compared with the other treatments. Compared with the control group, both CUNPs1.0a and CU1.5 treatments decreased (*p* < 0.01) the abnormality and dead sperm values. 

### 3.3. Effect of CU and CUNPs on Sperm Apoptosis in Post-Thawed Rabbit Sperm

The Results for sperm viable, early and late apoptosis and necrotic after cryopreservation are shown in Table 2. After thawing, the sperm viability was greater in CUNPs1.5 group than in the other groups. Overall, the supplementation of CU or CUNPs in any levels significantly enhanced the spermatozoa viability compared to the non-supplemented group. Additionally, post-thawing values using CUNPs (1.5 µg/mL) reduced in significantly values of early apoptotic, apoptotic and necrotic sperm in post-thawed rabbit semen compared to other groups. Furthermore, different supplementation levels of CUNPs from 0.5 to 1.5 µg/mL to tris-egg yolk extender enhanced (*p* < 0.001) sperm function through decreasing the percentages of apoptotic and necrotic sperms during semen cryopreservation.

### 3.4. Effect on Antioxidants Indices Post-Thawed Rabbit Sperm

The effects of CU and CUNPs on antioxidants indices of rabbit sperm after freeze-thawing are concise in Table 3. The greatest concentrations of TAC and GPx were recorded were for the CU1.5 group followed by CU1.0group, while the worst values for the control group. Moreover, the incorporation CUNPs (1.5 µg/mL) to tris-egg yolk extender improved TAC and GPx compared to the control and other CUNPs groups. It is interesting to notice that the supplementation of both levels (1 or 1.5 mg/mL) CUNPs to extender improved the activity of SOD followed by CU1.0 and CU1.5 groups. MDA and POC were lower in extenders containing both CU (1 or 1.5 µg/mL) and CUNPs (1.5 µg/mL) compared with the control extender (*p* < 0.05). The addition of CU at level 1.5 µg/mL to extender was recorded the lowest values of the lipid (MDA) and protein (POC) oxidations in comparison with other groups.

### 3.5. Effect on Sperm Ultrastructure in Post-Thawed Rabbit Sperm

**Figure 2:**
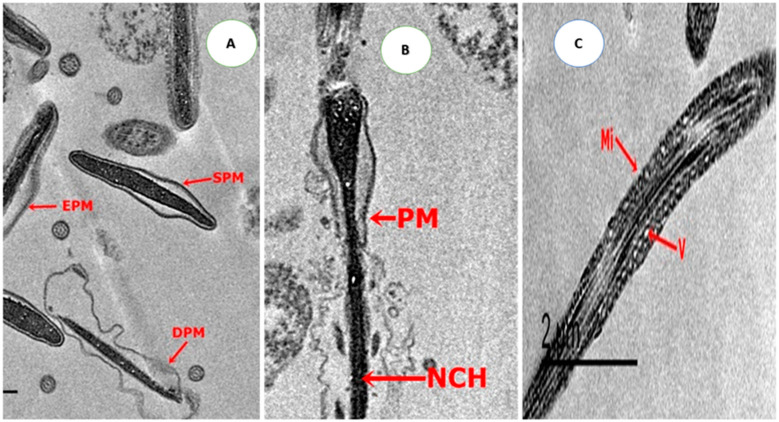
Transmission electron microscopic photomicrographs of sections of the rabbit sperm head in control, CU0.5, and CUNPs0.5 groups representing different stages of damage after freezing—thawing process. (**A**). The plasma membrane was slightly swollen (EPM) and damage in the plasma membrane (DPM); (**B**). A complete loss of PM, an abnormal nucleus with necrosis chromatin (NCH), damage of acrosomal cap (DAC), cell membrane and diffusion of acrosomal dense material into the previously formed space; (**C**). Abnormal mitochondrial sheath (Mi) appeared vacuolated (V) with narrowed membrane space and absence of cristae and cell membrane.

TEM micrographs of freeze-thawing semen showed various alterations ranging from slightly swollen until complete impairment (Figure 2) in the control and treated groups with 0.5 µg/mL of CU or CUNPs. An acrosomal cap separated from the nuclear membrane leaving a sub-acrosomal space and minor vesiculations also seemed, and the membrane was swallowed and may be lastly deteriorated. A scarce number of sperm cells presented entirely absent acrosome in the control, CU0.5, and CUNPs0.5 groups (Figure 2). Moreover, increasing mitochondria contents with distorted cristae were detected in sperm mid-piece. Impaired mitochondria performed vacuolated with narrowed membrane space (Figure 2). Generally, in the same head region, strong detachments of the plasma membrane with acrosome vesiculation were seen in frozen sperm. 

**Figure 3:**
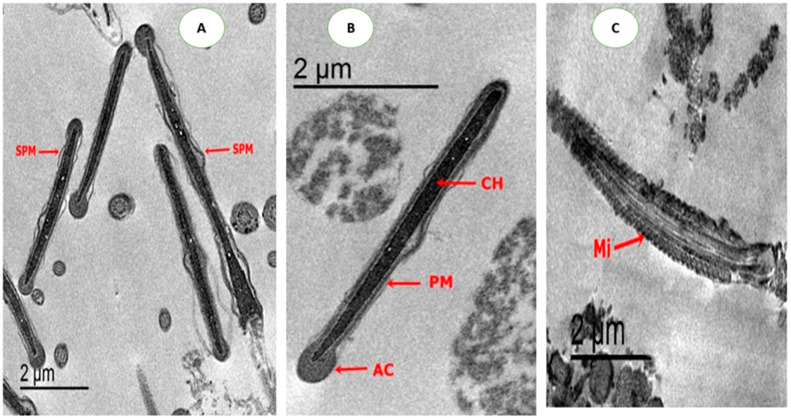
TEM photomicrographs of rabbit sperm head in groups treated with 1.0 µg/mL of CU or CUNPs. (**A**). A longitudinal section of head sperm showing normal nucleus and slightly extension of the cell membrane (SPM); (**B**). Intact plasma membrane (PM), apical ridge formation (AC), a normal nucleus with condensed chromatin (CH); (**C**). A mild degree of mitochondrial sheath damage was observed, and some regions have normal mitochondria.

**Figure 4:**
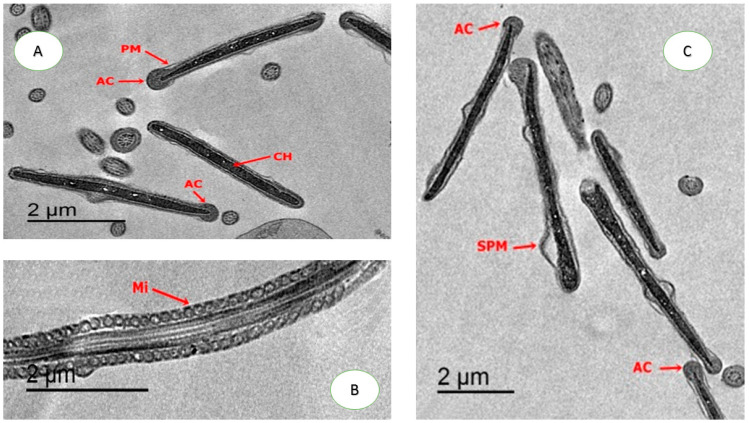
Transmission electron microscopic evaluation. Photomicrographs of sperm head in groups treated with 1.5 µg of CU or CUNPs. (**A**). Normal plasma membrane (PM) with intact head and apical ridge formation (AC) acrosomes and condensed chromatin (CH); (**B**). Early degree of cell membrane extension also was found (SPM) and normal apical ridge formation (AC) acrosomes; (**C**). Mitochondrion was regularly placed in longitudinal sections.

As shown in Figure 3. moderate damages in the head of sperm appeared in sperm cryopreserved with CU or CUNPs groups. Normal nucleus and apical ridge formation, and slightly extension of the cell membrane, was observed with nano-curcumin. Moreover, a slight degree of mitochondrial sheath damage and some regions have normal mitochondria were detected in both treatments CU1.0 and CUNPs1.0. Figure 4 shows normal membrane, condensed chromatin, apical formation, and mitochondrion is regularly placed in longitudinal sections were presented in CU1.5 and CUNPs1.5 groups.

## 4. Discussion

Over the last few decades, gamete cryopreservation technique has promptly progressed in animals for achieving successful fertilization via assisted reproductive technique. This technique helps preserve and transportation of valuable genetics for the fields of agriculture and biomedical research. During cryopreservation, spermatozoa are exposed to various stress which eventually leads to decreasing of the fertilizing capability of sperm. Hence, several records have engrossed on improving sperm quality and reducing the negative impacts of OS by adding of antioxidants [17] and nanoparticles [7,11,13,18], but no reports were conducted to use the curcumin nanoparticles in cryopreserved rabbit semen. It is well accepted that if the particles have low zeta potential values, then there will be no force to avoid the particles pending flocculating and together. The zeta potential of CUNPs was found to be −25 mV which presented strong stability of CUNPs. The imbalance between OS and antioxidants is detrimental to spermatozoa resulting in significant loss of function. CUNPs supplementation to rabbit semen extender at various tested concentrations was assessed for constraining the injurious impacts of OS synthesis, which damage the sperm cells during the freezing procedure. 

In light of this, several reports indicated that cryopreservation induced alteration in DNA integrity, lipid peroxidation, ROS, cholesterol content, capacitation [34], and acrosome reaction in spermatozoa and resulted in failure male fertility potential [5,7,17]. However, it has been reported that curcumin has a protective effect on DNA damage, normal development of testicular tissue and avoidance of spermatogenic cell apoptosis [12,15,18]. Studies have shown that CU has poor aqueous solubility, poor bioavailability, instability, rapid metabolism, and short half-life [18]. To improve these features; nanoparticles of curcumin have been developed for more efficiency, stability and bioavailability.

In this study, supplementing of CUNPs to the semen extender significantly enhanced motion criteria and plasma membrane integrity and reduced dead sperm and abnormality, but the greatest marks of sperm function were noticed with both groups CUNPs1.0 and CUNPs1.5. These improvements in sperm motion traits may be clarified by the improved antioxidant enzymes such as TAC, SOD, and GPx, as well as reducing oxidative induces (such as MDA and POC)—suggesting that CUNPs supplementation could improve the capability of seminal plasma to decrease the OS [13]. Besides, Raza et al. [35] clarified that CU can improve the activity of several defense enzymes, such as SOD and GSH in seminal plasma by sustained the mitochondrial redox signaling and respiratory functions. Khalil et al. [7] have demonstrated the addition of selenium nanoparticles 1.0 µg/mL in freezing medium, significantly improved sperm viability and motion features of Holstein bulls. Curcumin and curcumin nano-emulsion at high dose displayed significantly improved sperm quality (live sperm, mass and progressive motility) and reducing the dead and abnormality sperm in rats fed with protein-deficient diet [15]. The authors anticipated that CU has an anti-oxidative effect by decreasing the lipid and protein oxidation in the sperm cell membrane. Consequently, the CUNPs can mitigate OS injury through down-regulation of the H2O2 level and protect the spermatozoa from being injured by inflammatory factors, to achieve the purpose of protecting the sperm quality [36]. Furthermore, Falchi et al. [37] have revealed the antioxidant capability of cerium oxide (CeO2) nanoparticles on ram spermatozoa during storage at 4 °C. This might be related to the amounts of ROS were reduced within the semen extender through storage, which explained into better motion features and sperm viability. Supplementation of zinc oxide or selenium nanoparticles to the freezing medium had beneficial effects on post-thaw survival of human [38] and ram spermatozoa [10], respectively. 

Our data showed that reduction in viable spermatozoa, and the highest percentage of necrosis and apoptotic (early and late) in free extender without supplementation owing to the injurious effects of freezing that would cause evident spermatozoa membrane damage (Table 3). Prior investigation revealed that sterile men have reduced sperm functions induced by upper OS levels in semen [39]. They also confirmed that there are a positive association occurs between increased sperm impairment by OS and higher levels of caspases 9 and 3 and cytochrome c, which show positive apoptosis in patients with male issue sterility. Herein, we found significant improvement in the viability with CUNPs supplementation (1 or 1.5 µg/mL) and reduced significantly in apoptosis and necrosis percentages as measured by Annexin V/PI in rabbits. In line with our results, Zhang et al. [36] described that the level of the lipid peroxidation was significantly decreased in leucocytospermic patients treated with curcumin. Previous studies have shown that the supplementation of CU to Tris-yolk fructose extender significantly (*p* < 0.05) improved the viability and reduced (*p* < 0.05) percentages of necrotic and apoptotic sperm in frozen/thawed semen [17,18,40]. In sheep, it was found that dietary curcumin addition linearly repressed testicular apoptosis with augmented the expression of BCL and reduced the expression caspase-3 (*p* < 0.05) in testicular tissues [41]. It was indicated that nano-curcumin markedly decreased sperm abnormalities, the testicular tissue OS and apoptosis in rat testes fed with low protein in the diets [15]. It is on interest to note that the nano form of CU has been exhibited protective agent against the negative impacts of cryoinjury in rabbit frozen/thawed semen. In the other hand, despite the low bioavailability of curcumin in the crude form, enhancement of sperm functions was detected in the present study. 

Regarding the redox signaling in cryopreserved spermatozoa with nano-curcumin, it has been demonstrated that excessed OS are implicated in decreased fertilizing potential. Curcumin (1.5 µg/mL) appeared to be an effective dose for improving antioxidant enzymes; TAC, GPx, SOD followed by CUNPs (1.5 µg/mL). When antioxidant capacity is less than to remove excessive amounts of OS in semen, malondialdehyde (MDA) and protein carbonyl (POC) are generated caused intense cellular damage, and consequential accelerated apoptosis follows. Generally, these processes affect cell membrane integrity and DNA, resulting in cell death [3,42]. Enhancements of the antioxidant capacity and prevented or balanced the synthesis of OS are the main propose for improving the freezing potential of semen.

The present results indicated that beneficial effects of CU or CUNPs nanoparticles, at a high level (1 or 1.5 µg/mL) supplemented to the freezing medium, on post-thaw survival of rabbit spermatozoa via elevated the antioxidant capacity in seminal plasma. Hence, the nanoparticle has a promising strategy for using the maintenance of livestock sperm fertility during extended storage [16]. Besides, the use of POC as an indicator of oxidative stress has certain usefulness in contrast with the other oxidation parameters because of the relative early formation and relative stability of carbonylated proteins [42]. In our study, the POC levels could be reduced by crude curcumin, or curcumin nanoparticles supplementation. The lowest values of POC were detected in CU1.5, followed by CUNPs 1.5 and CU1.0 groups. Mostek et al. [3] stated that that cryopreservation process could bring oxidation of sperm proteins via carbonylation. These authors suggested that carbonylation of sperm proteins could be a direct result of free radicals and possibly lead to disturbances of capacitation-involved proteins or could exhibit cryopreservation-induced early capacitation [3]. In line with our results, Alizadeh et al. [19] have demonstrated that curcumin nano-micelle oral intake in human, resulted in a statistically significant enhancement in plasma levels of TAC, MDA, POC, and tumor necrosis factor. Curcumin administration induces upregulation of Nrf2, which might play a critical role to protect spermatozoa of asthenozoospermic through reducing ROS synthesis and apoptosis level [12].

The heterogeneity in sperm head dimensions appears a virtuous guide of sperm freezability in mammalian. So, based on morphometric characteristics, the presence of various sperm subpopulations is currently broadly recognized in many numbers of mammalian species, and it has been related to cryopreservation response [3]. Our TEM findings presented that the cryopreservation process tended to provide ultrastructure alteration in plasma membranes, acrosomal dense, apical ridge formation and the mitochondria in thawed-cryopreserved semen, leading to reduce the potential freezability. The present study found that the supplementation of nano-curcumin to the freezing medium in rabbit semen helps in alleviating the damage influence on the macromolecules like abnormal nucleus with necrosis chromatin, damage in the plasma membrane and crucial organelles like mitochondria. Additionally, transmission electron microscopic assessment exhibited some frozen sperm with disturbance of the acrosome membrane that might contribute to the reduction of sperm head size of some subpopulations in the control group compared with curcumin (1 or 1.5 µg/mL) and nano-curcumin (1 or 1.5 µg/mL) supplementation. The defensive role of CUNPs on sperm motion through the freezing/thawing process might be associated with the protective layer of CUNPs around the spermatozoa, which can avoid lipid or protein peroxidation in the sperm. Our results also revealed that CU or CUNPs supplementation, particularly at level (1 or 1.5 µg/mL) had a higher level of SOD and GPX and lower level of POC and MDA than the control, CU.05 and CUNPs0.5 groups, which reflect on semen quality. Curcumin in the nano form could counteract the negative influences of cryopreservation of rabbit semen by enhancing the antioxidant defense and reducing lipid or protein oxidations, thereby diminishing the oxidative stress. The current research offered a novel idea for discovering the use of nanotechnology against cryodamage.

## 5. Conclusions

Conclusively, the addition of curcumin and its nanoparticles to the extender can improve the progressive motility, viability, membrane integrity and sperm ultrastructure of post-thawed rabbit semen. These improvements might be associated with their capability to reduce the apoptosis, lipid and protein oxidations. The best improvement in the post-thawed rabbit semen quality was observed after using 1.5 µg/mL of CUNPs. These results encourage the application of nanotechnologies which could be easily involved in the semen processing and freezing protocols to enhance its quality. However, further studies are needed to evaluate the effects of adding different concentrations of curcumin and its nanoparticles on the conception and fertility rate of different animal species and to evaluate the biosafety of these nanoparticles to embryo, fetus, or body after fertilization.

## Figures and Tables

**Table 1:** Percentage of progressive motility, membrane integrity, viability and abnormality of post-thawed rabbit sperm as affected by graded levels of Curcumin (CU) and Curcumin nanoparticles (CUNPs) (mean ± SEM).

Parameters	Control	CU0.5	CU1.0	CU1.5	CUNPs0.5	CUNPs1.0	CUNPs1.5	SEM	*P*-Value
**Progressive motility (%)**	38.00 ^f^	53.00 ^e^	60.25 ^d^	63.50 ^c^	58.00 ^d^	68.00 ^b^	75.25 ^a^	0.966	<0.001
**Membrane integrity (%)**	44.20 ^f^	51.25 ^e^	62.50 ^c^	66.00 ^b^	55.50 ^d^	63.50 ^cb^	73.50 ^a^	0.985	<0.001
**Viability (%)**	46.00 ^a^	50.00 ^b^	45.50 ^c^	82.75 ^d^	56.75 ^c^	83.75 ^d^	71.75 ^e^	0.985	<0.001
**Abnormality (%)**	15.25 ^a^	15.00 ^a^	15.00 ^a^	12.75 ^b^	14.75 ^a^	12.00 ^b^	10.00 ^c^	0.381	<0.001

Control, no additive; CU0.5, CU1.0, and CU1.5, extender supplemented with 0.5, 1, and 1.5 µg/mL Curcumin; CUNPs0.5, CUNPs1.0, and CUNPs1.5, extender supplemented with 0.5, 1, and 1.5 µg/mL nanoparticles curcumin; ^a–f^ means with no common superscript within each row are differed significantly (*p* < 0.05).

**Table 2:** Effect of different levels of curcumin (CU) and curcumin nanoparticles (CUNPs) on sperm apoptosis in post-thawed rabbit sperm (mean ± SEM).

Parameters	Control	CU0.5	CU1.0	CU1.5	CUNPs0.5	CUNPs1.0	CUNPs1.5	SEM	*P*-Value
**Viable (%)**	30.40 ^f^	41.15 ^e^	46.85 ^d^	50.85 ^c^	45.90 ^d^	53.60 ^b^	62.30 ^a^	0.458	<0.001
**Early apoptosis (%)**	23.25 ^a^	15.00 ^b^	14.00 ^cb^	12.85 ^d^	13.60 ^cd^	12.75 ^d^	9.85 ^e^	0.335	<0.001
**Late apoptosis (%)**	25.65 ^a^	24.50 ^b^	20.45 ^d^	17.90 ^e^	22.10 ^c^	16.75 ^f^	12.30 ^g^	0.343	<0.001
**Necrosis (%)**	20.70 ^a^	19.35 ^b^	18.70 ^cb^	18.40 ^c^	18.40 ^c^	16.90 ^d^	15.55 ^e^	0.225	<0.001

Control, no additive; CU0.5, CU1.0, and CU1.5, extender supplemented with 0.5, 1, and 1.5 µg/mL Curcumin; CUNPs0.5, CUNPs1.0, and CUNPs1.5 extender supplemented with 0.5, 1, and 1.5 µg/mL nanoparticles curcumin; ^a–g^ means with no common superscript within each row are differed significantly (*p* < 0.05); SEM, standard error of least square mean.

**Table 3:** Effect of different levels of curcumin (CU) and curcumin nanoparticles (CUNPs) on antioxidants indices post-thawed rabbit sperm (mean ± SEM).

Parameters	Control	CU0.5	CU1.0	CU1.5	CUNPs0.5	CUNPs1.0	CUNPs1.5	SEM	*P*-Value
**TAC (ng/mg)**	14.85 ^g^	16.17 ^e^	19.72 ^b^	20.13 ^a^	15.73 ^f^	17.90 ^d^	19.00 ^c^	0.11	<0.001
**SOD (U/mg)**	32.91 ^e^	34.83 ^d^	44.75 ^b^	45.41 ^b^	38.58 ^c^	47.25 ^a^	48.66 ^a^	0.59	<0.001
**GPX (ng/mg)**	26.90 ^g^	31.38 ^e^	35.90 ^b^	38.62 ^a^	28.40 ^f^	33.67 ^d^	34.78 ^c^	0.28	<0.001
**MDA (nmol/mg)**	77.62 ^a^	55.25 ^c^	51.62 ^d^	34.25 ^e^	64.62 ^b^	55.00 ^c^	52.25 ^d^	0.63	<0.001
**POC (ng/mg)**	3.17 ^a^	2.26 ^c^	1.94 ^d^	1.52 ^e^	2.69 ^b^	2.28 ^c^	1.98 ^d^	0.04	<0.001

Control, no additive; CU0.5, CU1.0, and CU1.5, extender supplemented with 0.5, 1, and 1.5 µg/mL Curcumin; CUNPs0.5, CUNPs1.0, and CUNPs1.5 extender supplemented with 0.5, 1, and 1.5 µg/mL nanoparticles curcumin. TAC, total antioxidant capacity; SOD, superoxide dismutase; MDA, malondialdehyde; GPx, glutathione peroxidase; POC, protein carbonyl; ^a–b^ means with no common superscript within each row are differed significantly (*p* < 0.05); SEM, standard error of least square means.
